# Supplementary material for: Dynamic wrinkling pattern exhibiting tunable fluorescence for anticounterfeiting applications
Source: Nat Commun. 2020 Apr 14;11:1811. doi: 10.1038/s41467-020-15600-6 (PMC7156701; doi:10.1038/s41467-020-15600-6)
Supplement: Supplementary file 1 — Supplementary Information [file 41467_2020_15600_MOESM1_ESM.pdf]

SUPPLEMENTARY INFORMATION

Dynamic wrinkling pattern exhibiting tunable fluorescence for anticounterfeiting applications

Ma et al.

Supplementary Methods

Materials.

Styrene and n-butyl acrylate obtained from China National Pharmaceutical Group were washed by 5 wt% sodium hydroxide solution and then dried by anhydrous magnesium chloride. 4-vinyl pyridine purchased from TCI Chemical Co., Ltd, cyclopentanone obtained from Macklin Biochemical Co., Ltd, and ammonium acetate, 3,5-Bis(t-butyl)-4-hydroxybenzaldehyde and 3,5-Bis(t-butyl)benzaldehyde provided by Titansci Co., Ltd were used as received.

Synthesis of pyridine-containing polymer(P4VP-nBA-S)

The pyridine-containing polymer P4VP-nBA-S was synthesized by free radical copolymerization according to Supplementary Figure 1. 4-vinylpyride (1.85 g, 18 mmol), n-butyl acylate (4.50 g, 36 mmol)and Styrene (3.66 g, 36 mmol) were dissolved in 15 mL toluene at a feed molar ration 1:2:2, and then 100 mg 2,2-azobisisobutyronitrile (AIBN) was added (1 wt% total monomer weight). The polymerization reaction was performed at 70 °C for 12 hours under nitrogen protection. The reaction mixture was precipitated for three times in cold petroleum ether when cooled to room temperature. After the mixture was filtered, the product was dried at 70 °C for 24 hours. At last, we could obtain light yellow solid copolymer. The structure of P4VP-nBA-S was verified by ^{1}H NMR spectrum in Supplementary Figure 1. ^{1}H NMR (500 MHz, $CDCl_3$, δ ppm): 8.54-8.07 (CH-N-CH), 7.24-6.22 (benzene or pyridine ring), 4.12-3.35 (-O-CH_2-), 2.41-0.78 (-CH-CH_2-CH_3).

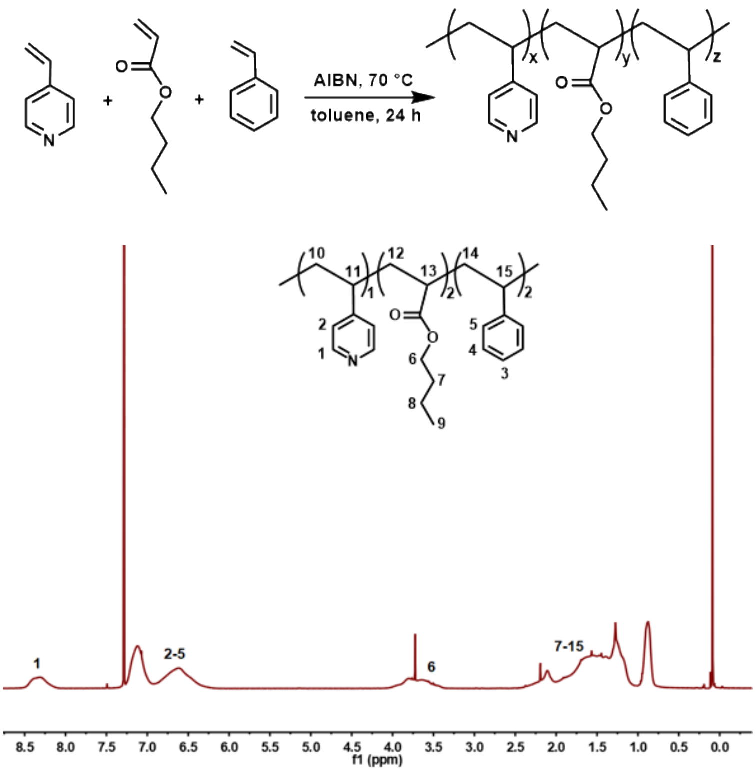

Supplementary Figure 1. The synthesis and ^{1}H NMR spectrum of P4VP-nBA-S.

Synthesis of hydroxy distyrylpyridine (DSP-OH)

The hydroxy distyrylpyridine (DSP-OH) was synthesized through one-step according to previous reports as shown in Supplementary Figure 2. A mixture of 3,5-Bis(t-butyl)-4-hydroxybenzaldehyde (23.4 g, 0.1 mol), cyclopentanone (4.21 g, 0.05 mol) and ammonium acetate (38.5 g, 0.5 mol) in ethanol (250 mL) with 1 mL of 30 % hydrogen peroxide was boiled during 1 h and left to stand overnight at 20 °C. The precipitate was refluxed in 50 mL acetone, and then filtered to get the product, which is dried in vacuum at 70 °C for 24 h. The structure of DSP-OH was verified by ^{1}H NMR spectrum in Supplementary Figure 2. ^{1}H NMR (500 MHz, $CDCl_3$, δ ppm): 7.67 (2H, =CH), 7.50 (4H, aromatic), 7.26 (2H, aromatic), 5.36-5.30 (3H, -OH), 3.19 (4H, $-CH_2-$), 3.07 (4H, $-CH_2-$), 1.70-1.25 (54H, $-CH_3$).

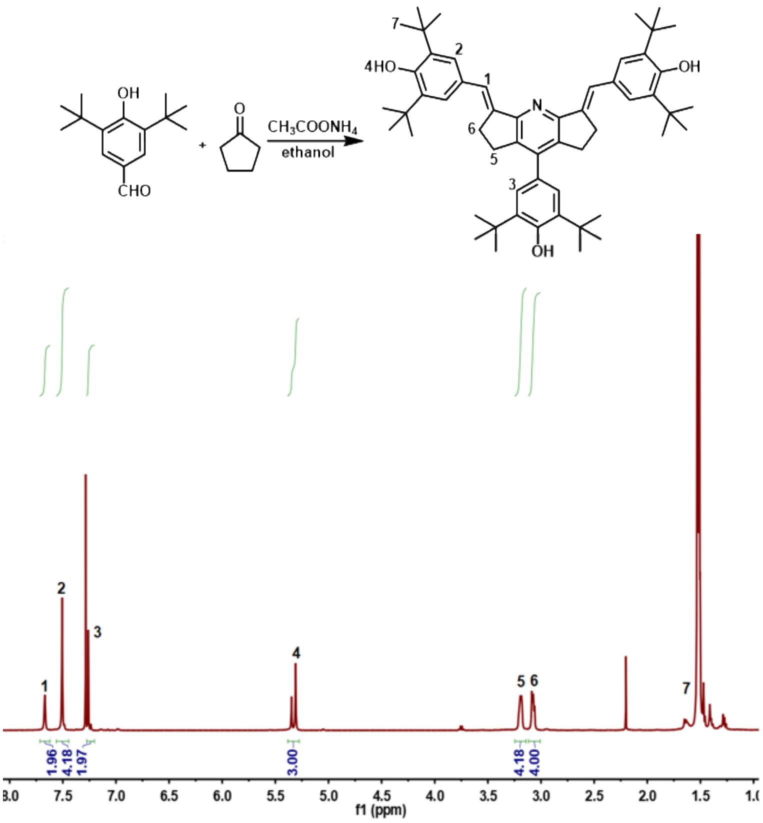

Supplementary Figure 2. The synthesis and ^{1}H NMR spectrum of DSP-OH.

Synthesis of t-butyl distyrylpyridine (DSP-Bu)

The t-butyl distyrylpyridine (DSP-Bu) was synthesized through one-step according to previous reports as shown in Supplementary Figure 3. A mixture of 3,5-Bis(t-butyl) benzaldehyde (4.37 g, 0.02 mol), cyclopentanone (0.84 g, 0.01 mol) and ammonium acetate (7.71 g, 0.1 mol) in ethanol (50 mL) with 0.2 mL of 30 % hydrogen peroxide was boiled during 1 h and left to stand overnight at 20 °C. The precipitate was refluxed in 10 mL acetone, and then filtered to get the product, which is dried in vacuum at 70 °C for 24 h. The structure of DSP-OH was verified by ^{1}H NMR spectrum in Supplementary Figure 3. ^{1}H NMR (500 MHz, $CDCl_3$, δ ppm): 7.81 (2H, =CH), 7.55 (4H, aromatic), 7.43-7.37 (3H, aromatic), 7.30 (2H, aromatic), 3.27 (4H, $-CH_2-$), 3.09 (4H, $-CH_2-$), 1.60-1.20 (54H, $-CH_3$).

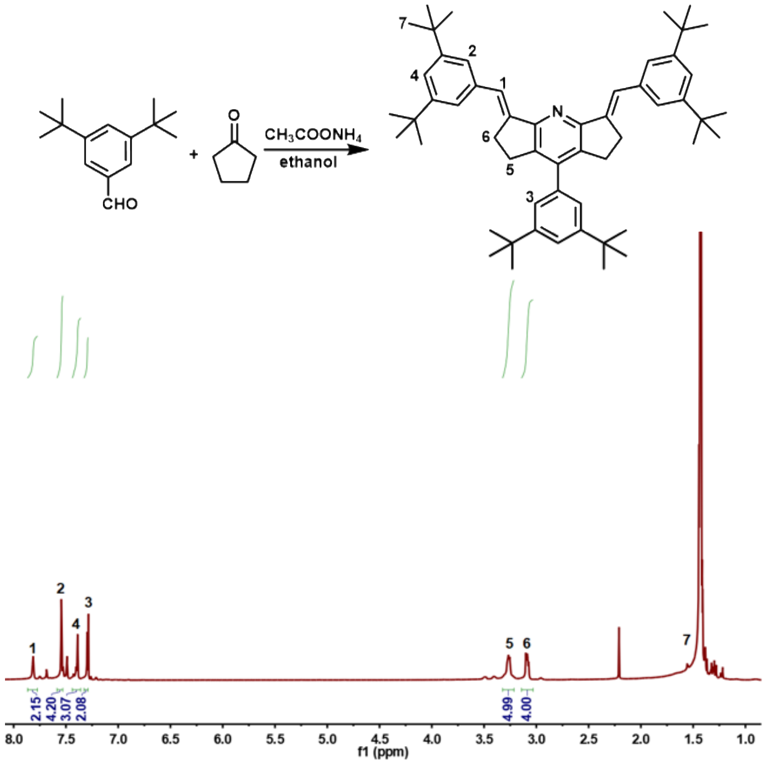

Supplementary Figure 3. The synthesis and ^{1}H NMR spectrum of DSP-Bu.

Preparation of PDMS Substrate

The PDMS elastic sheet was prepared by mixing PDMS prepolymer (Sylgard 184, Dow Corning) in a 10:1 base/curing agent ratio, followed by drop-coating in a Petri dish, degassing in a vacuum oven, and curing at 70 °C for 4 h (thickness approximately 400 μm). Then the sample was cut into 1 cm × 1 cm and 2 cm × 2 cm squares.

Preparation and erasure of Wrinkle Pattern

A toluene solution of P4VP-nBA-S(3 wt%) and DSP-OH(from 0.38 wt% to 1.5 wt%) was spin-coated onto a PDMS sheet to prepare the skin layer. The bilayer samples with fluorescent pattern were heated at 110 °C. When cooling to room temperature, wrinkled pattern occurred. As for erasure of the wrinkled and fluorescent pattern, the samples underwent 450 nm light or hydrogen chloride gas.

Characterization

^{1}H NMR spectra were recorded on a nuclear magnetic resonance (AVANCE III HD 500)

instrument with chloroform-d ($CDCl_3$) as the solvent and tetramethyl silane (TMS) as an internal standard at room temperature. Average molecular weights were determined by means of gel permeation chromatography (GPC, LC-20A, Shimadzu, Japan), using tetrahydrofuran as an eluent at a flow rate of 1.0 mL min^{-1} with a combination of two columns (Shodex, KF-802 and 804, 300 × 8 mm) and equipped with a RID-10A differential refractive index detector. The sample concentration was 2 mg mL^{-1} and the injection volume was 50 μL. Observation of the surface morphology and the measurement of the top-layer film thickness were performed by using an atomic force microscope(Dimension Icon & FastScan Bio, Bruker). Surface patterns were also recorded by profile measurement microscope (VF-7510, KEYNCE, Japan) and laser scanning confocal microscopy (LSCM, LEXT VK-X1000, Keyence, Japan). Infrared spectra were carried out by a Spectrum 100 Fourier transformation infrared absorption spectrometer (FT-IR, Nicolet IS10), which was recorded from 4000-400 cm^{-1} with a 4 cm^{-1} resolution over 32 scans. The isomerization process of DSP was traced by UV–Vis absorption spectrum using TU-1091 spectrophotometer (Persee, China). The fluorescence spectra were recorded using an LS-55B fluorescence meter (Perkin-Elmer, Inc., USA). The excitation wavelength is 365 nm. Fluorescence images (STED images) of coating were viewed with Super-Resolution Multiphoton Confocal Microscope (TCS SP8 STED 3X, Leica, Germany). The excitation wavelength is 405 nm. The UV and visible light source is LED and the intensity is 15 mW cm^{-2}.

Supplementary Discussion

The applied strain to trigger wrinkle formation in the bilayer system and the typical wavelength (λ) and amplitude (A)

According to linear buckling theory, the amplitude and wavelength of wrinkle is given as Supplementary Equations 1 and 2

$$A = h_{\mathrm{f}}\sqrt{\frac{\varepsilon-\varepsilon_{\mathrm{c}}}{\varepsilon_{\mathrm{c}}}} \quad (1)$$

$$\lambda = 2\pi h_{\mathrm{f}}\left(\frac{\bar{E}_{\mathrm{f}}}{3\bar{E}_{\mathrm{s}}}\right)^{2/3} \quad (2)$$

Here, ε_c refers to the critical strain to trigger wrinkle formation and is given as Supplementary Equation 3

$$\varepsilon_{\mathrm{c}} = \frac{1}{4}\left(\frac{3\bar{E}_{\mathrm{s}}}{\bar{E}_{\mathrm{f}}}\right)^{2/3} \quad (3)$$

By substituting Supplementary Equation 3 into Supplementary Equation 1, one obtains

$$\frac{A^2+h_{\mathrm{f}}^2}{h_{\mathrm{f}}^2} = \frac{4\varepsilon}{\left(\frac{3\bar{E}_{\mathrm{s}}}{\bar{E}_{\mathrm{f}}}\right)^{2/3}} \quad (4)$$

Both sides of Supplementary Equation 4 were taken as the natural logarithm and with the proper deformation, we can obtain Supplementary Equation 5 relating wrinkle amplitude A to the applied strain

$$\ln\left(A^2+h_{\mathrm{f}}^2\right) = \ln 4\varepsilon + \left(\ln 2h_{\mathrm{f}} + \frac{2}{3}\ln \bar{E}_{\mathrm{f}} + \frac{2}{3}\ln 3\bar{E}_{\mathrm{s}}\right) \quad (5)$$

Here, A, h_f, ε, and $\bar{E}$ represent the amplitude of the wrinkles, the thickness of the skin layer, the applied strain, and the plane-strain modulus, respectively, and the subscripts f and s refer to the skin layer and the substrate in the bilayer system.

Supplementary Figures

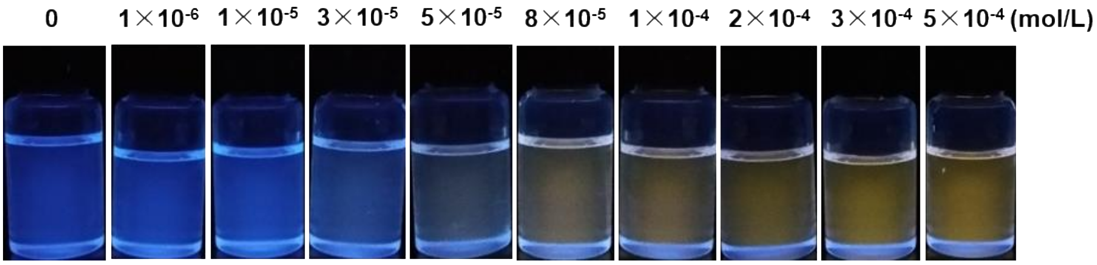

Supplementary Figure 4. The photographs of 1.0×10^{-6} mol L^{-1} DSP-OH toluene solution with different concentration of CF_3COOH taken under UV light.

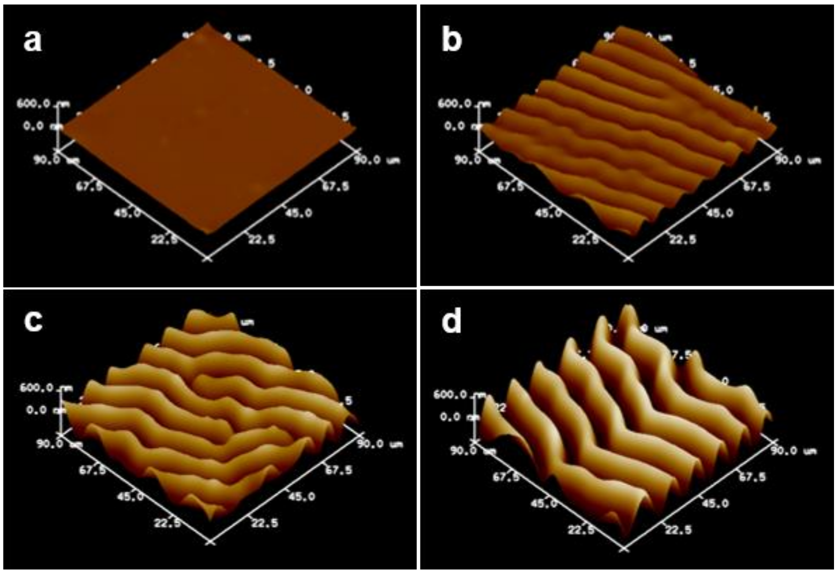

Supplementary Figure 5. 3D AFM images of wrinkled surface that P4VP-nBA-S(3 wt%) with different concentration of DSP-OH: **(a)** 0, **(b)** 0.38 wt%, **(c)** 0.75 wt%, **(d)** 1.5 wt%, respectively. Each sample underwent a thermal treatment at 110 °C. the 3D AFM images show growing amplitude and wavelength with increasing concentration of DSP-OH: (a) 0, 0; (b) 207 nm. 10.4 μm; (c) 612 nm, 12.3 μm; (d) 862 nm, 14.6 μm.

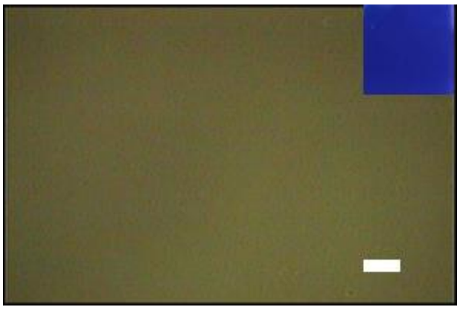

Supplementary Figure 6. Optical images of smooth surface that P4VP-nBA-S(3 wt%)/DSP-Bu(0.75 wt%) coated PDMS heated at 110 °C. Scale bar: 100 μm. Inset picture is the corresponding fluorescent photograph of sample taken under UV light.

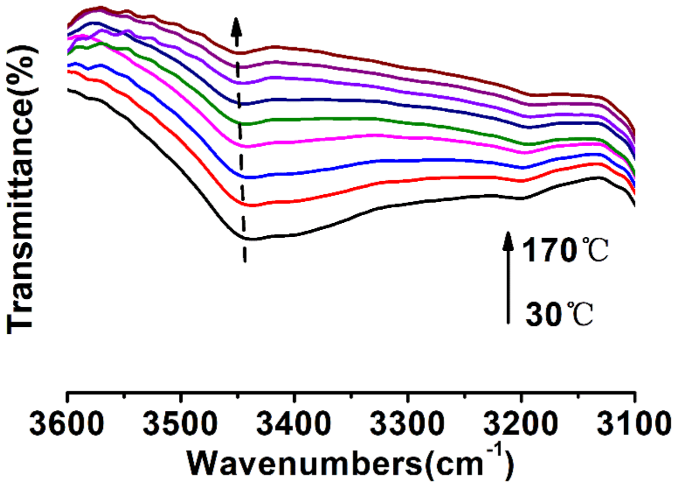

Supplementary Figure 7. Temperature dependent FT-IR spectra of P4VP-nBA-S/DSP-OH. The experimental temperature was 30, 40, 50, 70, 90, 110, 130, 150 and 170 °C, respectively.

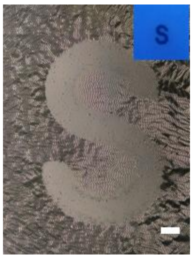

Supplementary Figure 8. LSCM images of “S” wrinkled pattern obtained by “S” mask. Inset picture is corresponding photograph of PDMS taken under UV light.

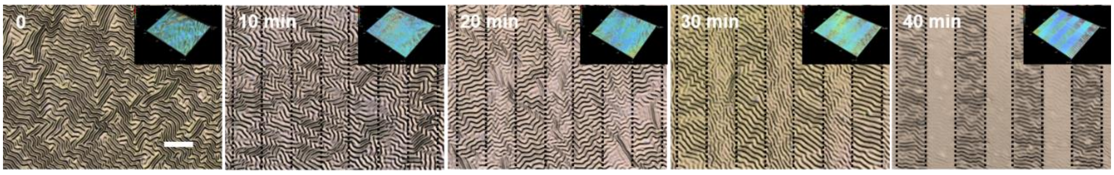

Supplementary Figure 9. LSCM images exhibiting the evolution of the wrinkled pattern irradiated by 450 nm light through a stripe photomask for different times: 0; 10 min; 20 min; 30 min; 40 min. Scale bar: 100 μm. Inset pictures are corresponding 3D LSCM images of the wrinkles.

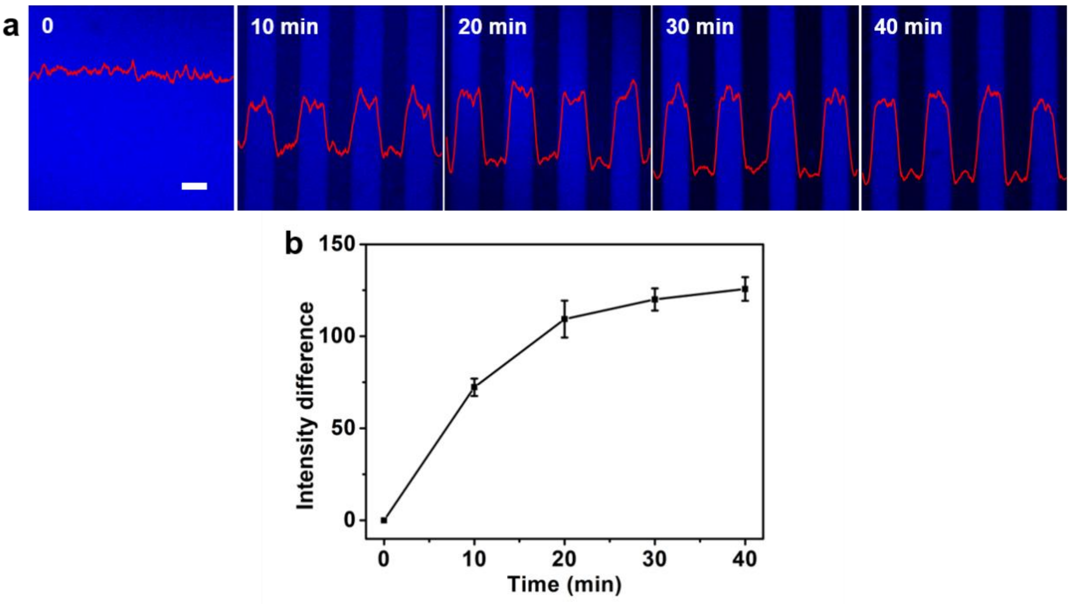

Supplementary Figure 10. (a) STED images exhibiting the evolution of the fluorescent pattern irradiated by 450 nm light through a stripe photomask for different times: 0; 10 min; 20 min; 30 min; 40 min. Scale bar: 100 μm. **(b)** The fluorescence intensity difference of exposed and unexposed regions as a function of 450 nm light irradiation time. Error bars represent the standard deviations of three independent data. Source data are provided as a Source Data file.

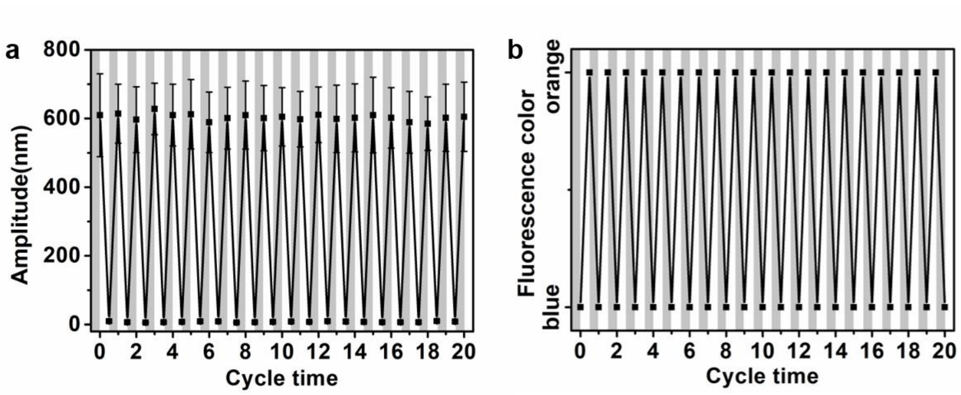

Supplementary Figure 11. (a) Process of erasure and recovery of wrinkles via exposure to the atmosphere containing HCl vapor. The white regions represented the erasure process exposed to the 56.4 ppm HCl vapor while the grey regions represented the recovery process through thermal treatment. Error bars represent the standard deviations of three independent data. Source data are provided as a Source Data file. **(b)** Process of fluorescence color change via exposure to the atmosphere containing HCl vapor. The white regions represented the erasure process exposed to the 56.4 ppm HCl vapor while the grey regions represented the recovery process through thermal treatment.

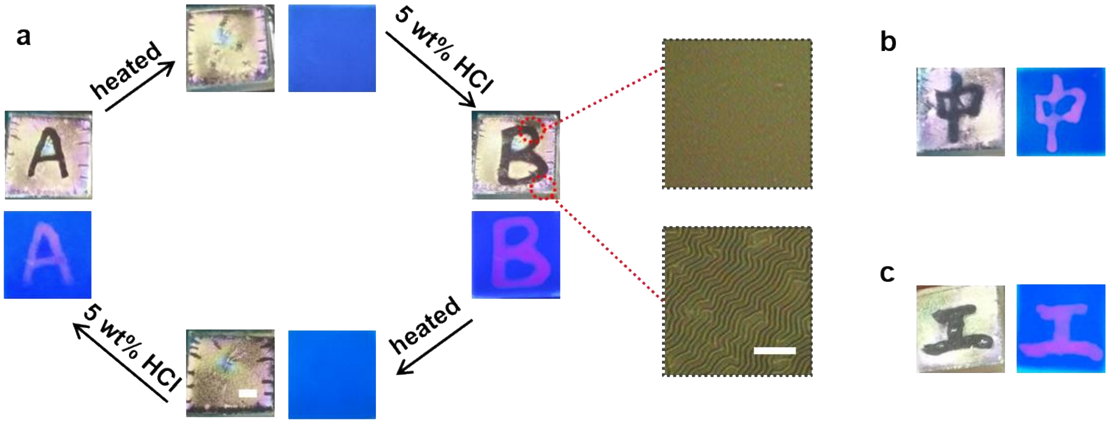

Supplementary Figure 12. Photographs of letters and Chinese characters on PDMS under UV light and natural light illustrating the application of the reversible dual-pattern in response to acid for smart displays. (a) Initial patterns with wrinkles and blue fluorescence, and the samples with the letters "A" and "B" written by a writing brush with 5 wt% HCl solution. **(b)** The Chinese character "zhong" which means "middle" written by a writing brush. **(c)** The Chinese character "gong" which means "work" written by a writing brush. Scale bar: 2 mm. The corresponding optical images exhibiting characteristic wrinkled and flat surface. Scale bar: 50 μm.

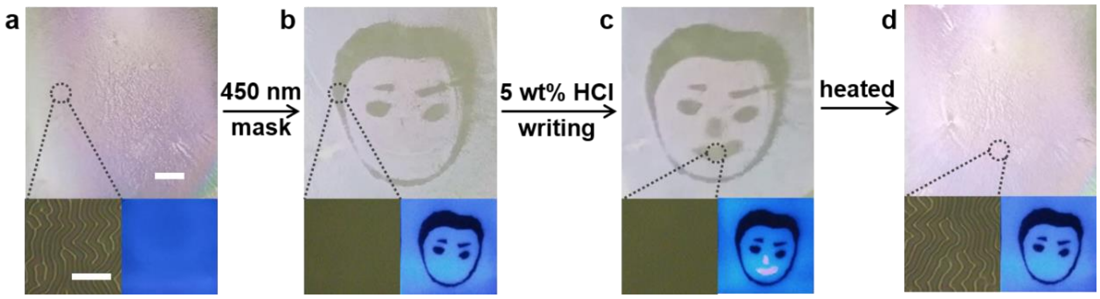

Supplementary Figure 13. Photographs of human face on PDMS under UV light and natural light illustrating the application of the multi-reversible dual-pattern for information storage and anticounterfeiting. (a) Initial labyrinth wrinkles and blue fluorescence. **(b)** Human face shaped wrinkled and blue fluorescent pattern without nose and mouth obtained by a photomask of human face under 450 nm light for 40 min. **(c)** Human face shaped wrinkled and blue fluorescent pattern with pink nose and mouth drawn by a writing brush with 5 wt% HCl. **(d)** Labyrinth wrinkled and human face shaped blue fluorescent pattern without nose and mouth that the sample (c) undergoing thermal treatment. Scale bar: 2 mm. The corresponding optical images exhibiting characteristic wrinkled and flat surface. Scale bar: 50 μm.

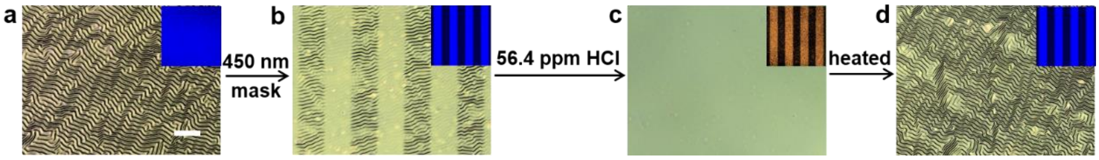

Supplementary Figure 14. LSCM and corresponding STED images of strip patterns with wrinkles and fluorescence illustrating the application for anticounterfeiting. (a) Initial labyrinth wrinkles and blue fluorescence. **(b)** Strip wrinkled and blue fluorescent pattern obtained by strip mask under 450 nm light for 40 min. **(c)** Flat and strip orange fluorescent pattern that the sample (b) undergoing 56.4 ppm HCl. **(d)** Labyrinth wrinkled and strip blue fluorescent pattern that the sample (c) undergoing thermal treatment. Scale bar: 100 µm.

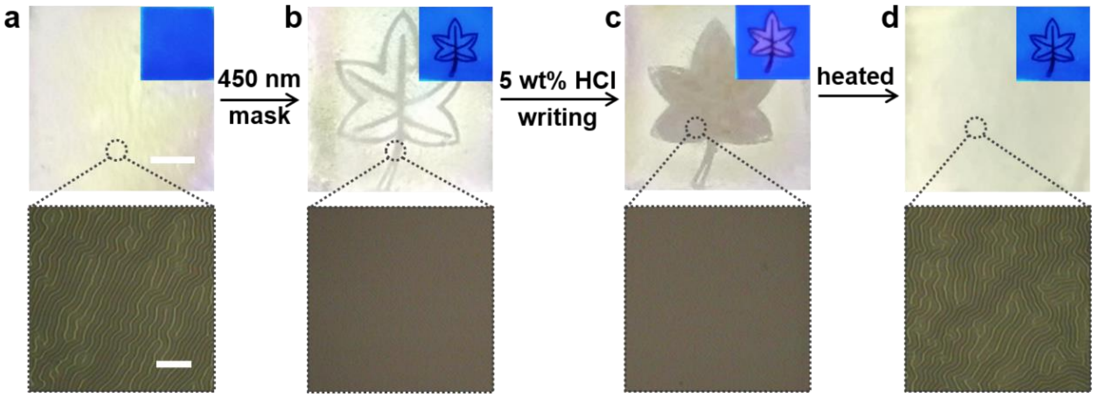

Supplementary Figure 15. Photographs of maple leaf on PDMS under UV light and natural light illustrating the application of the multi-reversible dual-pattern for anticounterfeiting. (a) Initial labyrinth wrinkles and blue fluorescence. **(b)** Leaf shaped wrinkled and blue fluorescent pattern obtained by a photomask of a maple leaf under 450 nm light for 40 min. **(c)** Leaf shaped wrinkled and pink fluorescent pattern drawn by a writing brush with 5 wt% HCl. **(d)** Labyrinth wrinkled and leaf shaped blue fluorescent pattern that the sample (c) undergoing thermal treatment. Scale bar: 2 mm. The corresponding images taken by microscope exhibiting characteristic wrinkled and flat morphology (down). Scale bar: 50 µm.
