# Supplementary material for: Dynamic wrinkling pattern exhibiting tunable fluorescence for anticounterfeiting applications
Source: Nat Commun. 2020 Apr 14;11:1811. doi: 10.1038/s41467-020-15600-6 (PMC7156701; doi:10.1038/s41467-020-15600-6)
Supplement: Supplementary file 3 — Description of Additional Supplementary Files [file 41467_2020_15600_MOESM3_ESM.pdf]

Description of Additional Supplementary Files

File Name: Supplementary Data 1
Description: In situ observation of the fluorescence color under 592 ppm HCl vapor as well as the wrinkled topography
